# Hospitalizations for rotavirus gastroenteritis in Gipuzkoa (Basque country), Spain.

**DOI:** 10.3201/eid0506.990619

**Published:** 1999

**Authors:** G Cilla, G E Pérez-Trallero, L D Piñeiro, A Iturzaeta, D Vicente


3. Chalmers RM, Salmon RL, Willshaw GA, Cheasty T, Looker N, Davies I, et al. Vero-cytotoxin-producing *Escherichia coli* O157 in a farmer handling horses. Lancet 1997;349:1816.
4. Chapman PA, Siddons CA, Cerdan Malo AT, Harkin MA. A 1-year study of *Escherichia coli* O157 in cattle, sheep, pigs and poultry. Epidemiol Infect 1997;119:245-50.
5. Sueyoshi M, Fukui H, Tanaka S, Nakazawa M, Ito K. A new adherent form of an attaching and effacing *Escherichia coli* (*eaeA+,bfp-*) to the intestinal epithelial cells of chicks. J Vet Med Sci 1996;58:1145-7.
6. Chapman PA, Siddons CA, Wright DJ, Norman P, Fox J, Crick E. Cattle as a possible source of verocytotoxin-producing *Escherichia coli* O157 infections in man. Epidemiol Infect 1993;111:439-47.
7. Akiba M, Masuda T, Sameshima T, Katsuda K, Nakazawa M. Molecular typing of *Escherichia coli* O157:H7(H-) isolates from cattle in Japan. Epidemiol Infect 1999;122:337-41.
8. Sekiya J. *Escherichia coli* O157:H7 in livestock in Japan. Revue Scientifique et Technique Office International des Épizooties 1997;16:391-4.
9. Ratnam S. March SB, Ahmed R, Bezanson GS, Kasatiya S. Characterization of *Escherichia coli* serotype O157:H7. J Clin Microbiol 1988;26:2006-12.

### Hospitalizations for Rotavirus Gastroenteritis in Gipuzkoa (Basque Country), Spain

**To the Editor**: Rotavirus is the main cause of severe acute gastroenteritis among children both in developing and in industrialized countries. The incidence of rotavirus gastroenteritis in northern Europe is similar to or greater than the estimated incidence of the disease in the United States (1-3); however, little is known about the impact of rotavirus infection on health in southern Europe.

We examined the incidence of hospitalization for rotavirus gastroenteritis during 3 years (July 1993-June 1996) in Gipuzkoa (population 400,480, of whom 58,896 are <15 years of age). The presence of rotavirus antigen was prospectively investigated by enzyme immunoanalysis (IDEIA Rotavirus, Dako Diagnostics, UK) in stool samples from all patients <15 years of age in the study area for whom a microbiologic analysis was requested for acute gastroenteritis. Children hospitalized for rotavirus gastroenteritis were sought retrospectively through searching both the computerized records of microbiology laboratory and hospital medical records for the diagnoses 558.9 (other gastroenteritis and presumably noninfectious colitis) and 008.6-009.3 (enteritis due to specific viruses and presumably infectious intestinal disorders) (4). All children in this study lived in the study area, had been hospitalized for gastroenteritis, and had one stool sample positive for rotavirus in the first 5 days of hospitalization without another gastroenteritis agent detected in the stool.

One hundred fifty-two (82 male and 70 female) of 1,004 children <15 years of age with rotavirus gastroenterititis had been hospitalized for rotavirus infection. No deaths were recorded. Cases usually occurred in epidemic waves, with the highest incidence in January-March. An additional 133 children with rotavirus in stools had been hospitalized but were not included in this study because they had hospital-acquired infections (67 cases), were coinfected by another microorganism (11 cases), came from outside the geographic study area (19 cases), or had a main reason for hospitalization other than gastroenteritis (36 cases). The mean annual incidence of hospitalization was 0.86 per 1,000 children (1 month to 14 years old) and 3.11 per 1,000 children (1 month to 5 years old). The maximum incidence occurred in 6- to 11-month-old children (11.81 per 1,000 children). Children were hospitalized for a mean of 4.8 ± 2.2 days. Rotavirus gastroenteritis was responsible for 152 (2%) of 7,403 pediatric admissions. For the 1- to 35-month age group, community-acquired rotavirus gastroenteritis was responsible for 140 (4.6%) of 3,026 admissions.

Although the incidence is based solely on confirmed cases, the findings closely reflect disease incidence in our region. The National System of Health covers 100% of the reference population, and hospitalization of children in private institutions is rare. Stool cultures were taken for most children for gastroenteritis (94.5%), and the presence of rotavirus was investigated in every case.

The hospitalization rate observed in this study was similar to that reported in other studies from Sweden (2), Denmark (5), and the United States (6) and lower than that found in England and Wales (3). In Spain, reporting of rotavirus infection is not required, is not included in mortality registers, and is not the object of specific vigilance by sentinel surveillance systems. Therefore, information about the incidence and impact of rotavirus infection in Spain is scarce. However, two reports from Spain must be highlighted: one is based on a theoretical prediction using a statistical model (7) and the other is a small clinical and epidemiologic study of hospitalized children <2 years of age in Santiago de Compostela (8). Data from both studies are consistent with our results. Rotavirus gastroenteritis is a common cause of hospitalization and produces a heavy load on the health-care system in our region. After years of research, vaccines that effectively prevent rotavirus infections in humans have been developed (9,10). These data should be considered in evaluating the potential benefits of introducing rotavirus vaccine in our region and monitoring its effectiveness.

### Acknowledgments

We thank Maribel Mendiburu and Antxon Nuñez for their valuable assistance.

This study was supported in part by a grant from the "Fondo de Investigaciones Sanitarias de la Seguridad Social" (Spanish Ministry of Health and Consumption), FIS 92/0612.

**G. Cilla G, E. Pérez-Trallero, L.D. Piñeiro, A. Iturzaeta, and D. Vicente**

Servicio de Microbiología, Complejo Hospitalario Donostia, San Sebastián, Spain

### References

1. Glass RI, Bresee JS, Parashar UD, Holman RC, Gentsch JR. First rotavirus vaccine licensed: Is there really a need? Acta Paediatr 1999;88 Suppl 426:S2-8.
2. Johansen K, Bennet R, Bondesson K, Eriksson M, Hedlund K-O, De Verdier Klingenberg, et al. Incidence and estimates of the disease burden of rotavirus in Sweden. Acta Paediatr 1999;88 Suppl 426:S20-3.
3. Ryan MJ, Ramsay M, Brown D, Gay NJ, Farrington CP, Wall PG. Hospital admissions attributable to rotavirus infection in England and Wales. J Infect Dis 1996;174 Suppl 1:S12-8.
4. Ministerio de Sanidad y Consumo. Clasificación internacional de enfermedades. Novena revisión. Modificación clínica, 1994.
5. Hjelt K, Krasilnikoff PA, Grauballe PC. Incidence of hospitalization and outpatient clinical visits caused by rotavirus and non-rotavirus acute gastroenteritis. A study of children living in the southern district of Copenhagen County. Dan Med Bull 1984;31:249-51.
6. Matson DO, Estes MK. Impact of rotavirus infection at a large pediatric hospital. J Infect Dis 1990;162:598-604.
7. Visser LE, Cano Portero R, Gay NJ, Martinez Navarro JF. Impact of rotavirus disease in Spain: an estimate of hospital admissions due to rotavirus. Acta Paediatr 1999;88 Suppl 426:S72-6.
8. Rodríguez-Cervilla J, Peñalver MD, Curros MC, Pavón P, Alonso C, Fraga JM. Rotavirus: Estudio clínico y epidemiológico en niños hospitalizados menores de dos años. An Esp Pediatr 1996;45:499-504.
9. Parashar UD, Bresee JS, Gentsch JR, Glass RI. Rotavirus. Emerg Infect Dis 1998;4:561-70.
10. Bernstein DI, Sack DA, Rothstein E, Reisinger K, Smith VE, O'Sullivan D, et al. Efficacy of live, attenuated, human rotavirus vaccine 89-12 in infants: a randomized placebo-controlled trial. Lancet 1999;354:287-90.

### Israeli Spotted Fever Rickettsia (*Rickettsia conorii* Complex) Associated with Human Disease in Portugal

**To the Editor:** Mediterranean spotted fever is endemic in Portugal, where it is a reportable disease with approximately 1,000 new cases per year (1). *Rickettsia conorii* has been thought to be the only pathogenic rickettsia of the spotted fever group in Portugal (2), as well as in the Western Mediterranean area. Another rickettsia in this group, the Israeli spotted fever rickettsia, which belongs to the *R. conorii* complex (3-5), was isolated in 1974 from ticks and humans; however, its distribution appeared to be restricted to Israel (6). We report three cases of rickettsiosis in Portugal caused by Israeli spotted fever rickettsia.

Case 1. A 71-year-old woman was hospitalized with a history of fever (39ºC) for 6 days, headache, and icterus. The influenzalike syndrome was treated with an antipyretic. In the next 4 days, the patient had myalgias, malaise, and mental confusion. Ten hours after being transferred to an intensive care unit, she died with septic shock and multiorgan failure, despite intravenous administration of doxycycline and other antibiotics.

Case 2. A 79-year-old woman, who was previously healthy except for high blood pressure, was hospitalized with a 4-day history of gastrointestinal disorders, nausea, and vomiting, which were attributed to food poisoning; high fever (40ºC) developed, and 3 days later a cutaneous rash, which spread to the palms and soles. The diagnosis of Mediterranean spotted fever was made by indirect immunofluorescent assay against *R. conorii* (immunoglobulin [Ig] M 1:40; IgG 1:512). The patient was treated with doxycycline and was discharged from the hospital 20 days after admission.

Case 3. A 65-year-old woman was hospitalized with a 6-day history of fever (39ºC), headache, vomiting, and epigastric pain, which had been treated with penicillin. Rash and

